# Assessing Neuronal and Astrocyte Derived Exosomes From Individuals With Mild Traumatic Brain Injury for Markers of Neurodegeneration and Cytotoxic Activity

**DOI:** 10.3389/fnins.2019.01005

**Published:** 2019-10-02

**Authors:** Charisse N. Winston, Haylie K. Romero, Maya Ellisman, Sophie Nauss, David A. Julovich, Tori Conger, James R. Hall, Wendy Campana, Sid E. O’Bryant, Caroline M. Nievergelt, Dewleen G. Baker, Victoria B. Risbrough, Robert A. Rissman

**Affiliations:** ^1^Department of Neurosciences, University of California, San Diego, La Jolla, CA, United States; ^2^Department of Anesthesiology, University of California, San Diego, La Jolla, CA, United States; ^3^Department of Pharmacology and Neuroscience, Institute for Translational Research, University of North Texas Health Science Center, Fort Worth, TX, United States; ^4^VA San Diego Healthcare System, La Jolla, CA, United States; ^5^Center of Excellence for Stress and Mental Health, La Jolla, CA, United States; ^6^Department of Psychiatry, University of California, San Diego, La Jolla, CA, United States

**Keywords:** traumatic brain injury, neuronal exosomes, astrocytes, amyloid, tau

## Abstract

Mild traumatic brain injury (mTBI) disproportionately affects military service members and is very difficult to diagnose. To-date, there is currently no blood-based, diagnostic biomarker for mTBI cases with persistent post concussive symptoms. To examine the potential of neuronally-derived (NDE) and astrocytic-derived (ADE) exosome cargo proteins as biomarkers of chronic mTBI in younger adults, we examined plasma exosomes from a prospective longitudinal study of combat-related risk and resilience, marine resiliency study II (MRSII). After return from a combat-deployment participants were interviewed to assess TBI exposure while on deployment. Plasma exosomes from military service members with mTBI (mean age, 21.7 years, *n* = 19, avg. days since injury 151), and age-matched, controls (deployed service members who did not endorse a deployment-related TBI or a pre-deployment history of TBI; mean age, 21.95 years, *n* = 20) were precipitated and enriched against a neuronal adhesion protein, L1-CAM, and an astrocyte marker, glutamine aspartate transporter (GLAST) using magnetic beads to immunocapture the proteins and subsequently selected by fluorescent activated cell sorting (FACS). Extracted protein cargo from NDE and ADE preparations were quantified for protein levels implicated in TBI neuropathology by standard ELISAs and on the ultra-sensitive single molecule assay (Simoa) platform. Plasma NDE and ADE levels of Aβ42 were significantly higher while plasma NDE and ADE levels of the postsynaptic protein, neurogranin (NRGN) were significantly lower in participants endorsing mTBI exposure compared to controls with no TBI history. Plasma NDE and ADE levels of Aβ40, total tau, and neurofilament light (NFL), P-T181-tau, P-S396-tau were either undetectable or not significantly different between the two groups. In an effort to understand the pathogenetic potential of NDE and ADE cargo proteins, neuron-like cultures were treated with NDE and ADE preparations from TBI and non-TBI groups. Lastly, we determined that plasma NDE but not ADE cargo proteins from mTBI samples were found to be toxic to neuron-like recipient cells *in vitro*. These data support the presence of markers of neurodegeneration in NDEs of mTBI and suggest that these NDEs can be used as tools to identify pathogenic mechanisms of TBI.

## Introduction

Traumatic brain injury (TBI) is a global public health concern. Epidemiological data suggests approximately 10 million people worldwide will sustain a TBI each year (Hyder et al., 2007). This is likely a gross underestimation as many mTBI cases go unreported, especially amongst sports athletes and military service members (Langlois et al., 2006). Approximately 75% of all TBI cases are mild TBI (mTBI) or concussions (Holm et al., 2005; Langlois et al., 2006). mTBI typically results in short-lived, neurological impairment that resolves spontaneously within 12 months (Binder, 1986; Karr et al., 2014). However, 5–20% of mTBI cases experience chronic, post concussive symptoms long after the injury (Ponsford et al., 2011; Karr et al., 2014; Eme, 2017). The underestimation of reported head injury cases and the chronic sequelae of TBI signifies the need for better diagnostic tools and therapeutic interventions for head injury patients.

Mild TBI diagnosis is complicated by the lack of interdisciplinary consensus regarding what constitutes a mTBI (Kulbe and Geddes, 2016; Prince and Bruhns, 2017). Because of this, the current criteria for mTBI can be difficult to assess in patients with pre-existing neurological conditions, intoxicated patients, and children (Kulbe and Geddes, 2016). In military populations, the recognition of mTBI is further complicated by the delay in diagnosis due to lengthy deployments (Marion et al., 2011). Many mTBI diagnoses are primarily based on the subjective nature of patients self-reporting mTBI symptoms and are likely to not be assessed until months or years after the initial impact (Kulbe and Geddes, 2016; Prince and Bruhns, 2017).

The current state of mTBI diagnostics includes expensive neuroimaging modalities (Lee and Huang, 2014; Shin et al., 2017) and cerebral spinal fluid (CSF) sampling (Agoston et al., 2017). Post concussive symptomology is insensitive to current neuroimaging techniques while CSF sampling is invasive and imposes an additional health risk to the patient. The urgent need to identify pre-symptomatic, neuropathological changes in the brain at earlier and more treatable timepoints has fueled research into biofluid biomarkers, specifically those obtained from plasma and serum. Extensive research has identified number of promising blood-based and CSF biomarkers of head injury including tau, neurofilament (NF), glial fibrillary acidic protein (GFAP), ubiquitin carboxyl-terminal hydrolase isoenzyme L1 (UCHL1), neuron-specific enolase (NSE), myelin basic protein (MBP), and calcium-binding protein (s100β). Many of these markers demonstrate high diagnostic accuracy for the acute stages of severe TBI (Agoston et al., 2017; Kim et al., 2018) while GFAP has recently emerged as demonstrating high diagnostic accuracy in differentiating mTBI patients from controls (Bogoslovsky et al., 2017). Work from our lab and others demonstrated the utility of neuronally derived, plasma exosomes (NDEs) as biomarkers for elucidating the neurodegenerative stages of AD and other diseases (Fiandaca et al., 2015; Goetzl et al., 2016a, 2018a; Hamlett et al., 2016; Stern et al., 2016; Winston et al., 2016; Ngolab et al., 2017). However, limited research has been conducted to assess the diagnostic potential of blood-based exosomes as a measurement of acute and chronic sequelae of mTBI (Gill et al., 2018; Goetzl et al., 2018c, 2019).

The acute and chronic sequelae of mTBI involves the activation of a diverse cascade of pathophysiological processes and events (DeKosky et al., 1998; Povlishock and Katz, 2005). Axonal damage, disruption of cytoskeletal networks along with the redistribution of neurofilament proteins are all thought to be the primary determinant of outcome following mTBI (Shahim et al., 2018) and severe TBI (Hamberger et al., 2003; Johnson et al., 2013). Because of this, many biofluid biomarker studies target cytoskeletal proteins including the microtubule stabilizing protein, tau and Neurofilament light (NFL). NFL is neuron specific, cytoskeletal protein found in myelinated axons. Axonal damage liberates tau and NFL from axons and releases it into the CSF and blood (Lin et al., 2018). Several longitudinal and prospective cohort studies support serum NFL as an ultrasensitive biomarker for mTBI, sports-related, repeat concussion (Shahim et al., 2017, 2018) and AD (Lin et al., 2018). Moreover, tau and phosphorylated epitopes of tau (p-tau) has been detected immediately after severe TBI (Tsitsopoulos and Marklund, 2013); plasma tau has been reported to be increased in military populations approximately 12 months after TBI (McKee et al., 2009; Rubenstein et al., 2015); and abnormal p-tau accumulation can be detected in post mortem brain many years after head injury (McKee et al., 2013; Tsitsopoulos and Marklund, 2013).

Neuroinflammation is also a well characterized pathological hallmark of head injury (Karve et al., 2016). Astrocytes, one of the major resident glial cells in the brain, were once thought to only provide structural support in the central nervous system (CNS) (Montgomery, 1994). Today, there is a greater appreciation for the diverse role that astrocytes play in the healthy CNS and following CNS insult and disease (Karve et al., 2016; Xiong et al., 2018). Previously, exosomes derived from astrocytes (ADEs) contained proteins associated with the generation of the toxic Aβ42 peptide (Goetzl et al., 2016b) and complement proteins that were associated with neurotoxic reactive astrocytes (Goetzl et al., 2018b; Winston et al., 2019). Similarly, cargo proteins of plasma ADEs accurately differentiated AD patients from age-matched controls (Goetzl et al., 2016b) and were found to be predictive biomarker of MCI conversion to AD (Goetzl et al., 2018b; Winston et al., 2019).

To examine NDE and ADE cargo proteins as biomarkers of chronic mTBI, we leveraged plasma samples from the Marine Resiliency Study II (MRSII), a prospective study of combat-related neurocognitive outcomes in 1,040 Marine and Navy service members (Glenn et al., 2017). Participants were assessed at pre-deployment and again at 4–6 months after returning from a combat deployment to Afghanistan for mental and physical health and cognitive performance. In the current study, we used plasma samples from 39 participants of this cohort (controls, *n* = 20; mTBI, *n* = 19), to isolate exosomes.

Exosomes were enriched by magnetic-bead immunocapture against the neural adhesion marker, L1CAM and the astrocytic marker, glutamine aspartate transporter (GLAST). Subsequently, all BAE (bead-antibody-exosome) preparations were FACS sorted. Protein cargo from NDE and ADE preparations were extracted, followed by quantitative determination of TBI-related markers via human specific ELISAs. The markers chosen were Aβ42, Aβ40, NFL, total tau, phosphorylated tau epitopes, T181 and S396, and calmodulin-binding, postsynaptic protein neurogranin (NRGN).

Absorption of NDE cargo from other neurodegenerative disorders are toxic to receipt cells *in vivo* (Winston et al., 2018), however, the pathogenic potential of plasma NDE and ADE cargo proteins from TBI samples has yet to be investigated. Lastly, we determined if cargo proteins from NDEs and ADEs were toxic to recipient cells *in vitro.*

## Materials and Methods

### Baseline Characteristics of Study Participants

Participants in this study, a subset of MRS-II participants, were all male, with a mean age at pre-deployment of 21.87 (SD = 2.76). On average assessments were given at 4 weeks (SD = 4.9) prior to deployment and again at 22 weeks (SD = 22.4) following deployment (Marine Resiliency Study II; Moore et al., 2017). MRS-II TBI assessment methodology mirrored that of MRS, which assessed life-time head injury (up to a maximum 5) at pre-deployment, and combat-related head injuries, defined as any head injury sustained between the pre-and post-deployment assessments (Yurgil et al., 2014). Detailed MRS methodology has been reported elsewhere (Baker et al., 2012); only descriptions of measures relevant to the present study are presented here. Demographic information (age, ethnicity, race) was collected via self-report surveys before deployment and was included in analysis as potential covariates. Head injury events were assessed via interview before deployment and after deployment. Interviewers gathered details of each reported injury, including injury cause or mechanism and symptom severity. TBI was defined as any head injury that resulted in loss of consciousness (LOC) or altered mental status (i.e., dazed, confused, or seeing stars, and/or posttraumatic amnesia (National Center for Injury Prevention and Control, 2003; von Holst and Cassidy, 2004; Helmick et al., 2006). Any head injury resulting in LOC and/or altered mental state (AMS; i.e., dazed, confused, “seeing stars,” and/or posttraumatic amnesia [PTA]) was defined as TBI (National Center for Injury Prevention and Control, 2003; O’Neil et al., 2014; Yurgil et al., 2014). For this study we selected post-deployment plasma samples from participants who self-reported multiple (2+) head injuries during the index deployment (*n* = 17 mean age, 21.74 ± 0.9; average number of TBI, 2.526 ± 0.1772, average number of days between most recent deployment TBI and sample collection 151 ± 112 days). In the TBI exposed group, 94% reported at least one injury that involved LOC, with the majority (82%) experiencing LOC < 15 min. Although the utility of neuroimaging has improved for mTBI diagnoses (Salat et al., 2017) imaging was not conducted on these participants. Moreover, no participant endorsed an injury with fracture or head wound. At the time of sample collection participants were asked if they were experiencing any current problems from the TBI, including memory problems, balance problems, headaches, sensitivity to light, irritability, and/or sleep problems. 94% endorsed experiencing at least one current symptom (average number of symptoms endorsed 3 ± 1.5). 76% of participants endorsed a blast/explosion-related TBI. Trauma- and deployment-exposed controls who did not endorse a history of TBI were selected for similarities in age, ethnicity/race, # of months in the military and range of trauma-symptoms as assessed by the Clinician Administered PTSD Symptom Scale (CAPS, version for DSM-IV) (Blake et al., 1995). The CAPS is a structured interview that is considered the gold standard for assessment of PTSD symptom severity. At the time of assessment, blood was drawn into EDTA-treated tubes, after which plasma was isolated for storage in −80°C freezers. See Demographics Table 1 for details.

**TABLE 1:** Demographics, military, and TBI history.

	**No history of TBI**	**TBI**
Age (years)	22.0 ± 1.2	21.7 ± 0.7
Race/Ethnicity	70% Caucasian, 5% African American, 15% Hispanic, 10% Asian/Other	71% Caucasian, 6% African American, 12% Hispanic, 11% Asian/Other
# Months in military	36.5 ± 12.1	32.9 ± 9.8
PTSD symptoms	23.25 ± 25	35.11 ± 25
Deployment stress	0.27 ± 0.61	0.79 ± 0.63^∗^
Average # of TBI	0	2.59 ± 0.8

*Measures are means ± SD. PTSD Symptoms assessed by Clinician Administered PTSD Symptom Scale for DSM-IV (Blake et al., 1995). Deployment Stress assessed using the Deployment Risk and Resiliency Inventory (variable is based on a composite score across four scales: post-battle experiences, combat experience, deployment concern, difficult living, and working environment (Vogt et al., 2013). ^∗^*p* < 0.05 vs. No history of TBI group, *t*_34_ = −2.55.*

This study was approved by the institutional review boards of the University of California, San Diego; the Veterans Affairs San Diego Research Service; and the Naval Health Research Center and written informed consent was obtained from all participants.

### Enrichment of Neuronal-Derived (NDEs) and Astrocyte-Derived Exosomes (ADEs) From Human Plasma via Bead Antibody Exosome (BAE) – FITC Complex and FACS Sort

Exosome isolation was conducted per manufacturer’s instructions (System Biosciences, Inc., Mountain view, CA, United States; Catalog # EXOQ5TM-1). Briefly, 250 μL of human plasma were incubated with 2.5 μL purified thrombin (System Biosciences, Inc.; Catalog # TMEXO-1) at room temperature for 5 min. After centrifugation at 10,000 rmp for 5 min, supernatants were incubated with 63 μL ExoQuick Exosome Precipitation solution (System Biosciences, Inc.; Catalog # EXOQ5TM-1) for 30 min at 4°C. Resultant suspensions were centrifuged at 1,500 × *g* for 1 h at 4°C. Supernatant was collected and the resultant pellet was suspended in 300 μL of 1× phosphate buffer saline (PBS) (diluted from 10× PBS; Thermo Fisher Scientific; Catalog# AM9625) with Halt protease and phosphatase inhibitor cocktail EDTA-free (Thermo Fisher Scientific; Catalog # 78443) and stored at −80°C until immunochemical enrichment of exosomes from both neural and astrocytic sources.

Neural and astrocyte enrichment was conducted per manufacturer’s instructions (System Biosciences, Inc.; Catalog # CSFLOWBASICA-1). Briefly, 40 μL of 9.1 μm, streptavidin magnetic Exo-Flow beads (System Biosciences, Inc.; Catalog # CSFLOWBASICA-1) were incubated with 100 ng/μL of mouse anti-human CD171 (L1CAM, neural adhesion protein) biotinylated antibody (clone 5G3, eBioscience/Thermo Fisher Scientific; Catalog # 13-1719-82) or mouse anti-human GLAST (ACSA-1) biotinylated antibody (Miltenyi Biotec, Inc., Auburn, CA, United States; Catalog # 130-118-984) for 2 h on ice, with gently flicking every 30 min to mix. Bead-antibody (Ab) complex was washed three times in Bead Wash Buffer (Systems Biosciences, Inc.; CSFLOWBASICA-1) using a magnetic stand. Bead-Ab complex was suspended with 400 μL of Bead Wash Buffer and 100 μL of total exosome suspensions rotating overnight at 4°C. Bead-Ab-exosome (BAE) complex was washed three times with Bead Wash Buffer then suspended in 240 μL of Exosome Stain Buffer and 10 μL of Exo-FITC Exosome FACS stain (Systems Biosciences, Inc.; Catalog # CSFLOWBASICA-1) for 2 h on ice, with gently flicking to mix. BAE-FITC complex was washed Three times in Bead Wash Buffer then suspended in 300 μL of Bead Wash Buffer prior to loading into BD FACS Aria II for sorting.

Flow-sorted, BAE-FITC complexes were incubated with 300 μL of Exosome elution buffer (System Biosciences, Inc.; Catalog # CSFLOWBASICA-1) at 25°C for 30 min. Finally, supernatant containing eluted exosomes were incubated with 1 μL of Exo-FlowIP clearing reagent (System Biosciences, Inc.; Catalog # EXOFLOW32A) at 37°C for 30 min then stored at −80°C.

### Quantification of NDE and ADE Protein Cargo by Human-Specific Enzyme Linked Immunosorbent Assays (ELISAs) and Using the Single Molecule Array (Simoa) Technology

Protein concentrations for eluted NDE and ADE suspensions were determined using the Pierce bicinchoninic acid (BCA) Protein Assay kit (Thermo Fisher Scientific; Catalog # 23225). Mammalian protein extraction reagent (M-PER) (Thermo Fisher Scientific; Catalog # 78501) with protease and phosphatase inhibitors are mixed with eluted NDE and ADE suspensions prior to ELISA quantification.

L1CAM-positive (NDE) cargo proteins and GLAST-positive (ADE) cargo proteins were quantified by human-specific ELISAs for P-T181-tau (Fujirebio US, Inc., Alpharetta, GA, United States; Catalog # 81582), Aβ1–42 (Cusabio, American Research Products, Waltham, MA, United States; Catalog # CSB-E10684h), P-S396-tau (Life Technologies/Invitrogen, Camarillo, CA, United States; Catalog # KHB7031), neurogranin (Cloud Clone Corp., American Research Products-Katy, TX, United States; Catalog # CEA404Hu), and tetraspanning exosome marker CD81 (Cusabio, American Research Products, Waltham, MA, United States; Catalog # CSB-EL004960HU) with verification of the CD81 antigen standard curve using purified human recombinant CD81 antigen (Origene Technologies, Inc., Rockville, MD, United States; Catalog # TP317508), according to suppliers’ directions. The mean value for all determinations of CD81 in each assay group was set at 1.00, and the relative values for each sample were used to normalize their recovery.

Neurofilament light, Aβ40 and total tau were measured using the Single Molecule Array (Simoa) technology (Simoa; Quanterix, Lexington, MA, United States) which enables detection of these biomarkers in human plasma, serum, CSF and enriched exosomes. The Simoa Human NF-Light Advantage kit (NFL) (Catalog # 103400) was used to determine the amount of NFL. The Simoa Human Neurology 3-Plex “A” (3-Plex) (Catalog # 101995) was used to quantitatively determine the amount of Ab42, Ab40, and Tau. Tests were performed to optimize dilution factors and centrifugation. 4× dilution factor was found to be suitable for the samples. After thawing, the samples were vortexed and spun at 10,000 g for 5 min; the supernatant was directly transferred to a 96 well plate (90 μL for singlet or 125 μL for duplicate). For NFL, a recombinant NFL calibration curve was constructed and transferred to the 96 well plate (334 μl). Calibration curve range was 0–500 pg/mL and with the dynamic range of 0–2000 pg/mL. Control samples (analog 200 pg/mL and digital 10 pg/mL) and inter-assay control (pooled normal plasma) were all transferred to the 96 well plate (90 μL for singlet or 125 μL for duplicate). The 96 well plate was loaded onboard and the desired dilution factor for the samples was created by the Simoa HD-1 analyzer. Utilizing a Two-step procedure in a reaction cuvette, samples were incubated with antibody coated paramagnetic beads and biotinylated antibody detector simultaneously. After a wash, streptavidin-conjugated β-galactosidase (SBG) reagent was added binding the biotinylated antibodies leading to SBG enzyme labeling of the captured NFL. After a second wash, the beads were re-suspended in resorufin β-D-galactopyranoside (RGP) reagent, transferred to a Simoa disk array and sealed. The NFL proteins captured by the antibody coated paramagnetic beads and labeled with the SBG reagent hydrolyze the RGP substrate to produce a fluorescence signal (Abs/Em = 573/585 nm). The fluorescent signal values generated from the calibration curve of known concentrations were fit using a 4-parameter logistic curve and 1/y^2^ weighting. The unknown samples and control samples concentrations were calculated from 4PL curve fit. Lower Limit of Detection of NFL was reported at 0.038 pg/mL and the Lower Limit of Quantification at 0.174 pg/mL.

Multiplexed detection of Aβ40 and total tau was accomplished by labeling beads with dyes of various wavelengths and concentrations creating distinct subpopulations of beads. Antibodies for each specific protein were immobilized to these color-encoded beads. Mixture of these beads were incubated with each sample generating detection of multiple proteins. From the materials provided, a recombinant 3-Plex calibration curve was constructed and transferred to the 96 well plate (334 μl). Calibration ranges for Aβ42, Aβ40, and total tau was 0–60, 0–140, and 0–100 pg/mL and dynamic ranges of 0–240, 0–560, and 0–400 pg/mL, respectively. Aβ40 and total tau control samples (analog 87.0, 393, and 99.5 pg/mL and digital 3.20, 22.4, and 2.24 pg/mL, respectively) and inter-assay control (pooled normal plasma) were all transferred to the 96 well plate (90 μL for singlet or 125 μL for duplicate). The 96 well plate was loaded onboard and the desired dilution factor for the samples was created by the Simoa HD-1 analyzer. A two-step process in a reaction cuvette was used and samples were incubated with antibody coated paramagnetic beads and biotinylated antibody detector simultaneously. After a wash, streptavidin-conjugated β-galactosidase (SBG) reagent was added binding the biotinylated antibodies leading to SBG enzyme labeling of the captured Aβ40 and total tau. After a second wash, the beads were re-suspended in resorufin β-D-galactopyranoside (RGP) reagent, transferred to a Simoa disk array and sealed. Aβ40 and total tau captured by the antibody coated paramagnetic beads and labeled with the SBG reagent hydrolyze the RGP substrate to produce a fluorescence signal (Abs/Em = 573/585 nm). As with NFL the fluorescent signal values generated from the calibration curve of known concentrations were fit using a 4-parameter logistic curve and 1/y^2^ weighting. The sample and control concentrations were calculated from 4PL curve fit. Lower limit of detection for Aβ40 and total tau was reported at 0.196 and 0.019 pg/mL, respectively, and the lower limit of quantification for Aβ40 and total tau was reported at 0.675 and 0.063 pg/mL, respectively.

### Differentiation and Exosome Treatment of PC12 Cells and Human Neuroblastoma SH-SY5Y Cells

PC12 cells were cultured in a 5% CO2 humidified atmosphere at 37°C. Cells were cultured in PDL-coated Sarstedt plastic T75 tissue culture flasks in DMEM supplemented with 10% horse serum and 5% fetal bovine serum. For exosome treatment, cells were transferred to poly-D-lysine (PDL)-coated, Olympus flat bottom, 12-well plates and plated at a density of 14,000 cells/well. Differentiation was induced by culturing cells in serum free media and 50 ng/ml NGF. After 48 h, PC12 cells showed signs of differentiation that included neurite sprouting. NDEs and ADEs preparations (100 ng/mL) from control and TBI samples were applied to NGF + PC12 cells and incubated for 48 h. Following the experiment, phase contrast images of PC12 cells were collected using a Nikon digital camera attached to a Zeiss Axiovert 35 microscope. Subsequently, cell culture media was collected and stored at −20°C.

Human neuroblastoma SH-SY5Y cells were maintained and differentiated as previously described (Kim et al., 2015). Briefly, cells were maintained in MEM:F12 + 10% FBS and incubated at 37°C in 5% CO2. For differentiation, cells were plated on rat tail collagen coated glass cover slips in MEM:F12 + 3% FBS supplemented with 15nM retinoic acid. Cells were refed every 2 days for 7 days for complete differentiation. NDEs preparations from control, TBI, and AD samples (100 ng/mL) were applied to SH-SY5Y cells and incubated for 48 h. Following the experiment, cell culture media was collected and stored at −20°C.

### Homogeneous Membrane Integrity Assay

CtyoTox-ONE^TM^ Homogeneous Membrane Integrity Assay was performed per manufactures instructions (Promega Corporation, Madison, WI, Catalog # G7890). Briefly, cell culture media from NDE and ADE-treated NGF + PC12 cells and SH-SY5Y cells were incubated with 2 μL of Lysis solution (per 100 μL original volume) for 25 min at 22°C and then aliquoted, in triplicate, into a 96 well assay plate. Equal volume of CytoTox-ONE^TM^ Reagent (100 uL) was added to each well containing cell culture media and incubated at 22°C for 10 min. 50 μL of stop solution was added prior to measuring fluorescent intensity at 562 nm using the iMark^TM^ Microplate Absorbance Reader (Bio-Rad Laboratories, Hercules, CA, United States).

### Statistical Analyses

Statistical significance of differences between means for cross-sectional groups mTBI and control were determined with an unpaired, non-parametric Mann–Whitney *t*-test (Prism 6; GraphPad Software, La Jolla, CA, United States). Receiver operating characteristic (ROC) analyses were conducted to assess the sensitivity of exosome cargo proteins in distinguishing among the two patient groups (Prism 6; GraphPad Software). ROC analyses were conducted under the non-parametric distribution assumption for standard error of area to determine the performance of classifier models (SPSS v21.0, IBM).

## Results

### Validating Neural and Astrocytic Enrichment of NDEs and ADEs From Human Plasma Using FACS Analysis

**FIGURE 1:**
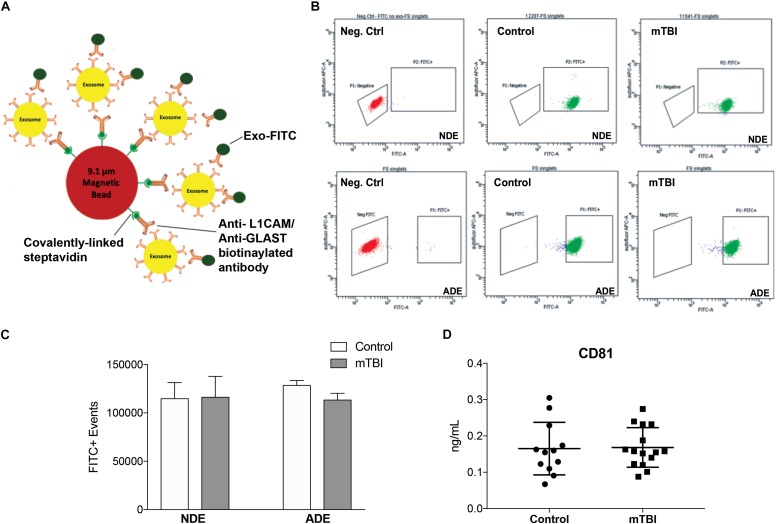
Fluorescent activated cell sorting (FACS) enrichment of plasma NDEs from service members with and without mTBI during deployment. Schematic cartoon of FACS enrichment via the formation of a bead-antibody-exosome (BAE) – FITC complex **(A)**. Representative FACS plot for non-exosome, negative control (red) and BAE – FITC complexes exosomes (green) isolated from control subject, 12207 (age: 22) and mTBI subject, 11541 (age: 21) and enriched against anti-human CD171 biotin (L1CAM) or anti-GLAST antibody **(B)**. Total number of FITC+ events were not significantly different between the two populations (**C**; 114719 ± 3712 FITC+ events vs. 116121 ± 4969 FITC+ events). Plasma NDE and ADE concentrations of exosome marker, CD81 were not statistically different between the two populations as measured by standard ELISA **(D)**.

A bead-antibody-exosome (BAE) – FITC complex was generated to enrich for neuronally derived (NDEs) and astrocyte derived (ADEs) exosomes from human plasma (Figure 1A). Mouse anti-human CD171 (L1CAM) or GLAST (ACSA-1) biotinylated antibody was coupled to magnetic streptavidin beads followed by the binding of plasma exosomes isolated from matched young, healthy military service members with no history of TBI and service members endorsing > 1 combat-related mTBI events within the past 4–6 months. The resultant BAE complex was coupled to a FITC fluorescent tag that only binds to exosomes (Exo-FITC, System Biosciences Inc.) and subsequently sorted based green fluorescent intensity. As a negative control, the resultant non-exosome, bead – antibody – FITC (BA – FITC) complex was sorted to confirm Exo-FITC secondary antibody only binds to exosomes (Figure 1B).

Fluorescent activated cell sorting analysis demonstrated that less than 0.5% of the particles are FITC-positive in the non-exosome, negative controls (BA-FITC) (Figure 1B) while the exosome preparations derived from control and TBI groups (Figure 1B) were between 76.4 and 99.8% Exo-FITC-positive (*n* = 19–20/group). The significant degree in flow separation from the non-exosome control demonstrates flow cytometry is an efficient method for validating neural and astrocytic enrichment of NDEs and ADEs from human plasma. The number of FITC positive (FITC+) events were quantified. We determined that the recovery of NDEs and ADEs following FACS were not significantly different between service members with mTBI and those with no TBI history (Figure 1C). Resultant exosome preparations were confirmed by ELISA against the exosome membrane protein marker, CD81 (Figure 1D). Similarly, plasma NDE levels for CD81 were not statistically different between the two patient populations (Figure 1D).

### Plasma NDE and ADE Levels of Aβ42 Are Increased While Plasma NDE and ADE Levels of NRGN Are Decreased in Young Service Members With Multiple mTBI Compared to Service Members With No TBI History

**FIGURE 2:**
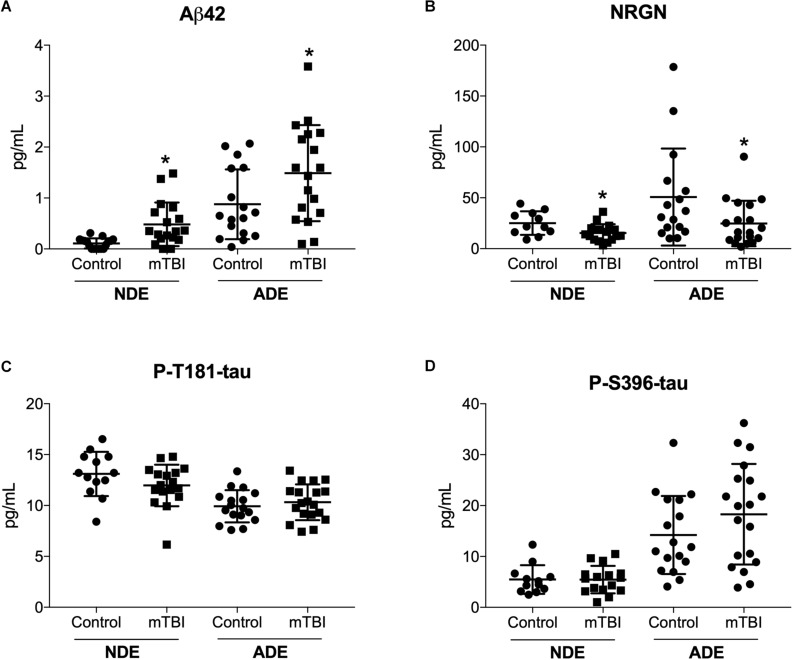
Human plasma NDE and ADE levels Aβ1–42 and NRGN differentiate young mTBI individuals from individuals with no TBI history. Plasma NDE and ADE **(A)** concentrations of Aβ42 were significantly higher, whereas plasma NDE and ADE **(B)** concentrations of NRGN were significantly lower in young individuals with mTBI (*n* = 17) compared to age-matched controls (*n* = 20). Plasma NDE and ADE concentrations of P-T181-tau **(C)**, and P-S396-tau **(D)** were not significantly different between the two groups. Non-parametric *t*-test, ^∗^*P* < 0.05 vs. control.

Previously, we reported plasma NDE levels of Aβ42, P-tau-T181, P-tau-S396 increased while plasma NDE levels of NRGN decreased in MCI (mean age, 68.7 years) and AD (71.1 years) patients as compared to age-matched controls (mean age, 70.8 years) (Winston et al., 2016, 2019). Here, we observed that plasma NDE and ADE levels of Aβ42 (Figure 2A, *P* < 0.05) were significantly increased and levels of NRGN (Figure 2B, *P* < 0.05) were significantly decreased in mTBI samples as compared to age matched controls with no TBI history. Plasma NDE and ADE levels of P-T181-tau were not significantly different between the two groups (Figure 2C). Plasma NDE levels of P-S396-tau were not significantly different between the two groups while plasma ADE levels of P-S396-tau were elevated in the mTBI group as compared to controls, however, these data failed to reach significance (Figure 2D). Across groups, ADE cargo showed higher levels of Aβ42 (Table 2, 1.18 pg/mL ADEs vs. 0.29 pg/mL NDEs) and P-396-tau (Table 2, 16.26 pg/mL ADEs vs. 5.47 pg/mL NDEs) compared to plasma NDE cargo. All NDE and ADE concentrations for biomarkers analyzed were normalized against CD81 (Figure 1D).

**TABLE 2:** Plasma NDE and ADE protein quantification as measured by ELISA and the Sioma platform.

**NDE – Hu ELISA**			**ADE – Hu ELISA**		
**Protein Cargo**	**Control pg/ml ± S.E.M.**	**mTBI pg/ml ± S.E.M**	**Protein Cargo**	**Control pg/ml ± S.E.M.**	**mTBI pg/ml ± S.E.M**

Aβ42	0.11 ± 0.02	0.48 ± 0.10^∗^	Aβ42	0.88 ± 0.17	1.49 ± 0.22^∗^
NRGN	25.13 ± 3.49	15.45 ± 2.16^∗^	NRGN	50.77 ± 11.92	24.74 ± 5.11^∗^
P-T181-tau	13.10 ± 0.60	11.97 ± 0.49	P-T181-tau	9.93 ± 0.39	10.32 ± 0.40
P-S396-tau	5.48 ± 0.81	5.45 ± 0.68	P-S396-tau	14.22 ± 1.86	18.30 ± 2.27

**NDE – Quanterix Simoa**			**ADE – Quanterix Simoa**		

**Protein Cargo**	**Control pg/ml ± S.E.M.**	**mTBI pg/ml ± S.E.M**	**Protein Cargo**	**Control pg/ml ± S.E.M.**	**mTBI pg/ml ± S.E.M**
Aβ40	0.99 ± 0.19	0.75 ± 0.18	Aβ40	0	0
Total Tau	1.55 ± 0.17	1.69 ± 0.18	Total Tau	0.13 ± 0.01	0.12 ± 0.01
NFL	1.47 ± 0.17	1.57 ± 0.14	NFL	0.02 ± 0.01	0.02 ± 0.01

*Quantitative determination of NDE and ADE cargo proteins were conducted using an unpaired, non-parametric Mann–Whitney *t*-test. Plasma NDE and ADE concentrations of Aβ42 were significantly higher, whereas plasma NDE and ADE concentrations of NRGN were significantly lower in young individuals with mTBI (*n* = 17) compared to age-matched controls (*n* = 20). ^∗^*P* < 0.05. Cross sectional analysis revealed that plasma concentrations of ADE cargo (control + mTBI) were significantly higher for Aβ42 (1.18 pg/mL ADEs vs. 0.29 pg/mL NDEs, *P* < 0.05) and P-396-tau (16.26 pg/mL ADEs vs. 5.47 pg/mL NDEs; *P* < 0.01) as compared to plasma concentrations of NDE cargo (control + mTBI).*

**FIGURE 3:**
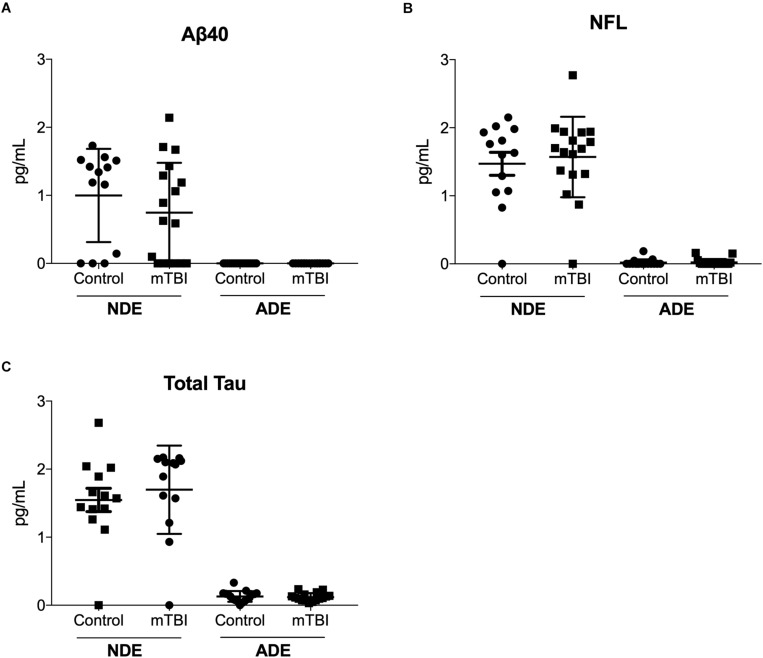
Plasma NDE levels of Aβ40, total tau, and NFL are not significantly different between individuals with and without mTBI. Plasma NDE levels of Aβ40 **(A)**, NFL **(B)**, and total tau **(C)**, were not significantly different between the two populations while plasma ADE Aβ40 **(A)**, NFL **(B)**, and total tau **(C)** were not detectable by the Simoa Assay.

Quantitative determination of NDE cargo proteins on the Simoa platform revealed plasma NDE levels of Aβ40 (Figure 3A), NFL (Figure 3B), and total tau (Figure 3C) were not significantly different between the two patient populations while plasma ADE levels Aβ40, total tau, and NFL were negligible or undetectable on the Simoa platform.

### Plasma NDE Cargo Protein From Service Members With mTBI Are Toxic to Recipient Cells *in vitro*

Lastly, we determined the pathogenic potential of plasma NDE and ADE cargo protein *in vitro*. The neuronal-like characteristics of differentiated PC12 cells are well documented (Greene and Tischler, 1976) and serve as an excellent *in vitro* model system for the study of neuronal function and survival. Differentiated PC12 cells (NGF + PC12 cells) were incubated with 100 ng/mL of NDE or ADE preparations for 48 h (averaged triplicates; *n* = 4 individual samples/group). Exosome-induced cytotoxicity was assessed by measuring the amount of lactate dehydrogenase (LDH) released into the cell culture media. LDH is a soluble protein that is found in all living cells. The release of LDH into the surrounding media is a result of compromised membrane integrity and a marker of cell death.

**FIGURE 4:**
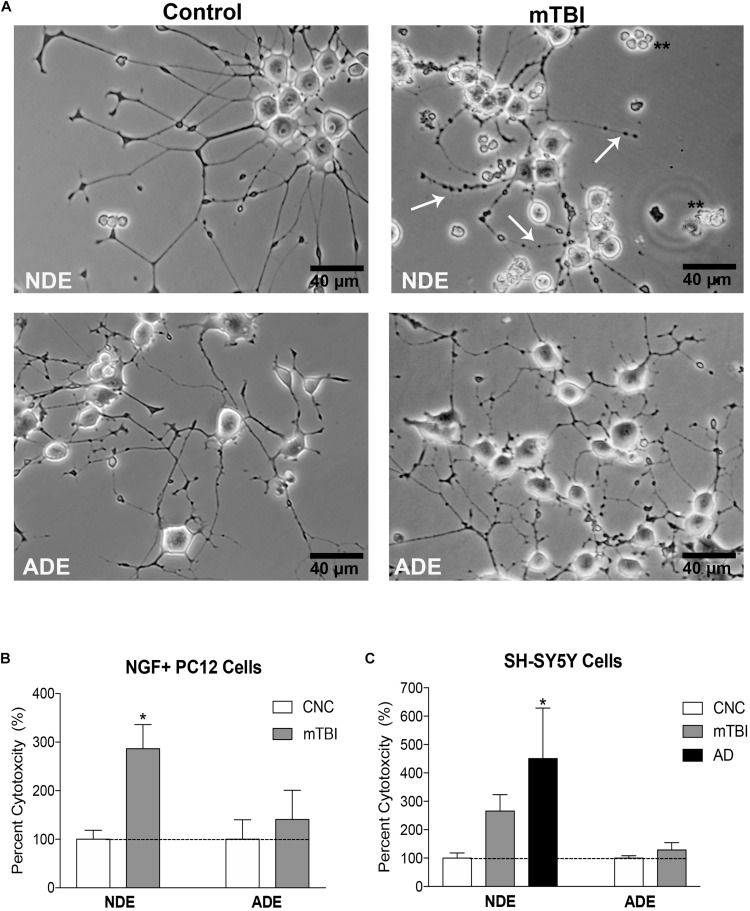
Plasma NDE cargo from individuals with mTBI compromise membrane integrity and are toxic to neuron-like cells *in vitro*. Differentiated PC12 cells and SH-SY5Y cells were incubated with 100 ng/ml of NDE and ADE for 48 h. Representative photomicrographs indicate exosome-induced cytotoxicity. Signs of cytotoxicity include dendritic blebbing (indicated by arrows), suppressed neurite outgrowth and cell death in NGF + PC12 cells **(A)**. Compared to controls (100% viability), there was a threefold increase in exosome-induced cytotoxicity (LDH release) in wells that were treated with NDEs derived from service members with mTBI **(B)**, ^∗^*P* < 0.05 vs. control, dashed horizontal line represents 100% viability. SH-SY5Y cells were treated with NDE and ADE preparations from control and mTBI service members and AD patients. A similar level of cytotoxicity was observed in cultured SH-SY5Y. A threefold increase in exosome-induced cytotoxicity (LDH release) was observed in wells that were treated with NDEs derived from mTBI samples and a fourfold increase exosome-induced cytotoxicity in wells that were treated with NDEs derived from AD samples as compared to NDEs derived from control samples **(C)**. Non-parametric *t*-test; ^∗^*P* < 0.05 vs. control, dashed horizontal line represents 100% viability.

After 48 h, wells treated with plasma NDEs from mTBI individuals displayed overt signs of cytotoxicity. Representative photomicrographs depicted signs of dendritic blebbing, suppressed neurite outgrowth, and cell death (Figure 4A). Compared to controls (100% viability), there was approximately threefold (286.7%) increase in exosome-induced cytotoxicity in wells that were treated with NDEs derived from mTBI individuals (Figure 4B, *P* < 0.05).

Next, we aimed to replicate our findings in a different *in vitro* model system. Differentiated human neuroblastoma SH-SY5Y cells were treated with 100 ng/mL NDE or ADE preparations for 48 h (averaged triplicates; *n* = 4 samples/group). Treatment of SH-SY5Y cells with plasma NDE preparations from patients diagnosed with AD was included as a positive control. Again, we observed approximately threefold (265.8%) increase in exosome-induced cytotoxicity in wells that were treated with NDEs derived from samples of service members with mTBI. In wells that were treated with NDEs derived from AD patients, we observed approximately fourfold (450.3%) increase in exosome-induced cytotoxicity as compared to NDEs derived from controls (Figure 4C, *P* < 0.05). Interestingly, plasma ADE cargo proteins from service members with mTBI did not induce cytotoxicity or compromise the membrane integrity of NGF + PC12 cells or SH-SY5Y cells (Figures 4B,C).

Together, these data suggest that plasma NDE cargo proteins from individuals exposed to multiple recent (within 4–6 months) mTBI events are toxic to neuron-like cells *in vitro*. Furthermore, plasma exosomes from different cell types mediate divergent biological processes in the CNS.

## Discussion

In the current study, we report, for the first time, that plasma NDE and ADE levels of Aβ42 and NRGN can differentiate military service members with mTBI from those with no TBI with moderate sensitivity and accuracy (see ROC analysis, Table 3). Because of the matched control strategy these differences are unlikely to be due to age, race/ethnicity, # months in military or PTSD symptoms. Plasma NDE and ADE levels of Aβ42 were significantly increased (Figure 2A) while plasma NDE and ADE levels of NRGN (Figure 2B) were significantly decreased in the group with combat-related mTBI, as compared to those without mTBI. Plasma ADE levels of P-S396-tau were elevated in the mTBI group as compared to controls, however, these findings failed to reach significance (Figure 2D). Interestingly, plasma NDE and ADE levels of Aβ40, P-T181-tau, total tau, and NFL were either undetectable or not significantly different between the two groups.

**TABLE 3:** Receiver Operating Curve analysis for biomarker sensitivity for cargo proteins measured by standard ELISA.

**NDE**			**ADE**		
**Protein cargo**	**Sensitivity (% ±SEM)**	**Confidence interval (%)**	**Protein cargo**	**Sensitivity (% ±SEM)**	**Confidence interval (%)**
A042	84.6 ± 0.7	70.1–98.4	Aβ42	69.4 ± 0.08	51.8–87.1
NRGN	74.4 ± 0.9	55.1–93.7	NRGN	71.1 ± 0.09	53.9–88.1

Given that TBI can initiate the abnormal accumulation of Aβ and tau (Tsitsopoulos and Marklund, 2013), it was surprising to see that plasma NDE and ADE levels of total tau, P-T181-tau, P-S396-tau were not significantly different between the two groups. Surprisingly, cross sectional analysis revealed that total plasma concentrations of ADE cargo (control + mTBI) were significantly higher for Aβ42 (see Table 2; *P* < 0.05) and P-396-tau (see Table 2; *P* < 0.01) as compared to total plasma concentrations of NDE cargo (control + mTBI). It’s not well understood why total levels of for Aβ42 and P-396-tau were higher in ADEs as compared to NDEs. Studies have demonstrated that astrocytes undergo phagocytosis in response to damaged neighboring cells (Liddelow and Barres, 2017; Wakida et al., 2018). Thus, its plausible that injury induced accumulation of Aβ42 and P-396-tau activated a phagocytic response in astrocytes. This response could result in higher levels of said proteins within astrocytes that are subsequently exocytosed and trafficked to the periphery via ADEs. Future studies include determining how exosome biogenesis and function differ between various cell populations.

Here, we detect changes in protein levels contained within exosomes. However, many of the previous studies reported elevated levels of Aβ42, total tau and p-tau in serum and/or CSF. One recent study reported elevated levels of exosomal p-tau and total tau in individuals who sustained > 3 mTBI injuries as compared to healthy controls and to those who sustained 2 or fewer mTBIs (Kenney et al., 2018). Interestingly, Kenney et al. (2018) reported Aβ42 and Aβ40 were undetectable in total exosomes derived from military veterans and controls. Gill et al. (2018) extracted NDEs from plasma and reported tau, Aβ42 and IL-10 were elevated in military servicemembers who had a mTBI and experienced a LOC of 20 min or less. In each of these studies, including the current study, biomarker quantification was assessed in patients with varying injury severity (mild to moderate); the number of self-reported mTBI varied amongst the study participants; some participants reported LOC while others did not; and the source of biomarker quantification also varied (total exosomal content vs. NDEs/ADEs). Understanding how all of these factors impact the expression of various TBI-related, exosome cargo proteins in the blood will aid in developing better mTBI diagnostics, prognostics, and would further elucidate mTBI pathophysiology.

Retrospective cohort studies confirm the relationship between age, injury severity, and outcome following TBI (LeBlanc et al., 2006). The age of onset is likely another factor that impacts the utility of blood-based, exosome biomarkers for mTBI. Our study participants were 10–20 years younger than the aforementioned studies (Gill et al., 2018; Kenney et al., 2018) and upward of 40–50 years younger than study participants of other exosome-related, biomarker studies (Fiandaca et al., 2015; Winston et al., 2016, 2019). It’s not fully understood why certain proteins are packaged into exosomes and trafficked into the periphery, however, the temporal profile of blood-based biomarkers may vary across different age groups. Exosomes are known to shuttle cargo, including proteins and micro RNA (miRNA) between cells and aid in the removal of excess or damaged, intracellular proteins and their cellular contents (Rajendran et al., 2006; Kalani et al., 2014; Budnik et al., 2016). However, the basic mechanisms behind exosome transport and function in the healthy and diseased CNS are still unknown (Hessvik and Llorente, 2018). In order to enhance the clinical utility of exosomes in therapy and diagnosis, it is essential that we gain a better understanding of these cellular processes (Hessvik and Llorente, 2018). Moreover, creating a profile with age-appropriate NDE and ADE biomarkers will also enhance the diagnostic and prognostic utility of blood-based exosome biomarkers for mTBI.

The risk of developing of chronic traumatic encephalopathy (CTE) is a growing concern for military personnel and sports athletes (Randolph et al., 2013; Chauhan, 2014; Tartaglia et al., 2014). CTE is a slowly progressing neurodegenerative disease that typically affects those who suffer repeat mTBI over extended periods of time. CTE is a clinically and neuropathologically distinct tauopathy that can only be diagnosed post mortem (McKee et al., 2009). The ultimate concern, especially in younger patients, is continued exposure of repeat-mTBI during the latency period. Identifying blood-based, exosome biomarkers for mTBI would also serve is useful prognostic biomarkers for the progression of mTBI to CTE (Stern et al., 2016). Recently, Goetzl et al. (2019) quantified NDE cargo proteins in young sports athletes (mean age, 20.67 years) who sustained repeat-mTBI acutely (7 days) and chronically (3–12 months) prior to the time of blood donation. They determined plasma NDE levels of Aβ42 and P-T181-tau were elevated in acute and chronic mTBI patients while plasma NDE levels of P-S396-tau were elevated only in chronic mTBI patients as compared to controls (Goetzl et al., 2019). These data suggest that the diagnostic potential of the blood-based, exosome cargo proteins, Aβ42 and p-tau, are sensitive to varying inter-injury intervals (latency period between mTBI). In our study, blood collection occurred 4–6 months after the service members returned from deployment. These differences may also help explain why we didn’t observe a significant difference in plasma NDE and ADE levels of p-tau and total tau between the two patient populations.

Interestingly, we observed no difference in plasma NDE levels of NFL between the two patient groups despite numerous studies suggesting serum and CSF NFL is a highly sensitive biomarker for concussion (Shahim et al., 2017, 2018). Shahim et al. (2017) reported levels of serum NFL levels were elevated between 7–10 days after a bout in young amateur boxers (mean age, 21.5 years), however, after 3 months of rest, serum NFL levels were significantly decreased in the amateur boxers (Shahim et al., 2017). Although, very few studies have quantified NFL in exosomes (Kawata et al., 2018), it’s likely that NFL may only serve as an acute biomarker for mTBI. Plasma ADE levels of NFL (and Aβ40) derived from the two groups were undetectable. It’s plausible that the release of NFL from the neuronal cytoskeleton may not be readily taken up by astrocytes, or these markers are immediately degraded following astrocyte absorption.

It’s evident by these recent findings that there is not a single, “one-size fits all” biomarker suitable for mTBI diagnosis. The discrepancies across all of these studies call into question the approach that has been used to identify blood-based biomarkers for mTBI. The handful of previously identified biomarkers were derived from studies that screened biofluids for markers that were known to be associated with severe TBI pathophysiology (Agoston et al., 2017). This approach has limited the success for blood-based biomarker diagnostics for mTBI as the etiology for both conditions are not the same. Using an unbiased discovery approach, to identify and generate a panel with multiple blood-based biomarkers that are unique to mTBI and that accurately represent mTBI pathophysiology will likely be more efficacious than assessing the sensitivity of a single biomarker.

The ability to detect neurological biomarkers in biofluids, other than CSF, and at very low levels has also been challenging and has further limited the utility of blood-based biomarkers for mTBI. Moreover, the hesitancy to use exosome cargo as biomarkers for mTBI is derived from the lack of conclusive evidence that altered levels of exosome cargo proteins accurately reflect disease pathogenesis in the CNS. Exosomes have been shown to act as nano-surrogates of their cells of origin and possess unique signatures which reflect the status of the cell. Therefore, using CNS-derived exosomes to quantify levels of protein biomarkers may be more relevant than free circulating protein levels in whole blood.

Lastly, we investigated the pathogenetic potential of plasma NDE and ADE exosome cargo proteins in neuron-like cells. Work from our lab and others demonstrated that NDEs can propagate toxic proteins *in vivo* (Reilly et al., 2017) and induce toxicity to recipient cells *in vitro* (Winston et al., 2018). In the current study, we confirmed that plasma NDEs are toxic to recipient cells, and we report, for the first time that plasma ADEs were not toxic to neuron-like cells. Plasma NDEs and ADEs both contained detectable levels of Aβ42 and p-tau, however, only the cargo proteins from plasma NDEs of individuals with mTBI were toxic to neuron-like cells *in vitro*.

One explanation is ADEs are not easily taken up by neuronal-like cells. Alternatively, the reduced concentration of Aβ42 and p-tau in plasma ADEs as compared to plasma NDEs were insufficient to induce cytotoxicity. It’s also plausible that plasma ADEs contained anti-inflammatory cytokines and other trophic molecules that attenuated cytotoxicity in recipient cells. We hypothesize that NDEs and ADEs have dual neuroprotective and neurotoxic roles in the CNS. One study reported that exosomes from astrocytes release inflammatory cytokines, including IL-1β, to induce an inflammatory response (Kalani et al., 2014) while another study determined that neuronal exosomes contain proteins, such as Ndfip1 and Nedd4, which are thought to play an important role in removing toxic proteins after injury (Putz et al., 2008). We acknowledge the limitations of our cytotoxicity assays which include the use “neuron-like” cells vs. primary neuronal and astrocyte cultures. Despite these limitations, we report similar results in two distinct cell culture model systems. Future studies will include primary cultured cells from neurons and astrocytes and exosome treatment in pre-clinical animal models. Lastly, levels of complement proteins (Winston et al., 2019) and pro- and/or anti-inflammatory cytokines were not quantified in the current study, however, that is also future direction. Addressing this in future studies will provide better insight into the dual role that plasma exosomes derived from astrocytes may play in mTBI and other neurodegenerative diseases. Further investigation is needed to determine how exosomes from different cells mediate divergent biological processes in the healthy CNS and during CNS injury and disease.

Additional limitations to the study should also be considered. This was a male, military sample and thus may not be generalized to other forms of mTBI in civilians or in women. Imaging and helmet sensor verification for the injuries was not available thus case-control selection was based on self-report, a common problem for military mTBI studies. Indeed, retrospective self-report is most commonly all that is available for clinical TBI diagnoses in the Veteran population, and thus identification of potential biomarkers through exosome cargo may yield important future clinical applications. Studies are now ongoing to identify the timecourse of these proteins following injury and to disentangle if these proteins predict long-term chronicity and/or comorbidity with other disorders such as PTSD and neurodegenerative disorders in Veterans that endorse prior TBI.

## Data Availability Statement

All datasets generated for this study are included in the manuscript/supplementary files.

## Ethics Statement

The studies involving human participants were reviewed and approved by UCSD and VA San Diego. The patients/participants provided their written informed consent to participate in this study.

## Author Contributions

CW and RR designed the study. CW evaluated the patients. CW, ME, SN, HR, DJ, TC, and JH performed the laboratory work. CW, RR, VR, and SO wrote the manuscript. WC helped design the present study, specifically the in vitro assays. VR initiated and helped to design the present study and supervised the original sample collection study (MRSII) and sample selection. DB administratively oversaw MRS data collection activities. CN and DB supervised the collection and storage of the samples and associated demographic, health, and TBI data, and edited the manuscript.

## Conflict of Interest

The authors declare that the research was conducted in the absence of any commercial or financial relationships that could be construed as a potential conflict of interest.
